# Distraction from pain depends on task demands and motivation

**DOI:** 10.1097/PR9.0000000000001041

**Published:** 2022-10-26

**Authors:** Todd A. Vogel, Carl F. Falk, A. Ross Otto, Mathieu Roy

**Affiliations:** Department of Psychology, McGill University, Montreal, QC, Canada

**Keywords:** Pain, Distraction, Motivation, Attention, Rewards, Cognition, *N*-back

## Abstract

**Introduction:**

Pain captures attention automatically, yet we can inhibit pain when we are motivated to perform other tasks. Previous studies show that engaging in a cognitively demanding task reduces pain compared with a task that is minimally demanding, yet the effects of motivation on this pain-reducing effect remain largely unexplored.

**Objectives:**

In this study, we hypothesized that motivating people to engage in a task with high demands would lead to more cognitive resources directed toward the task, thereby amplifying its pain-reducing effects.

**Methods:**

On different trials, participants performed an easy (left–right arrow discrimination) or demanding (2-back) cognitive task while receiving nonpainful or painful heat stimuli. In half of the trials, monetary rewards were offered to motivate participants to engage and perform well in the task.

**Results:**

Results showed an interaction between task demands and rewards, whereby offering rewards strengthened the pain-reducing effect of a distracting task when demands were high. This effect was reinforced by increased 2-back performance when rewards were offered, indicating that both task demands and motivation are necessary to inhibit pain.

**Conclusions:**

When task demands are low, motivation to engage in the task will have little impact on pain because performance cannot further increase. When motivation is low, participants will spend minimal effort to perform well in the task, thus hindering the pain-reducing effects of higher task demands. These findings suggest that the pain-reducing properties of distraction can be optimized by carefully calibrating the demands and motivational value of the task.

## Abstract 2

Supplemental Digital Content is Available in the Text.

Distraction is effective at reducing pain when the task is both cognitively demanding and there is sufficient motivation to invest effort into the task.

## 1. Introduction

Pain captures attention and compels us to protect ourselves,^15^ yet it can often be ignored to prioritize other tasks. According to Fields' motivation–decision model,^10^ pain can be inhibited when a competing action is more strongly motivated.^37^ Other studies have shown that performing a competing task can distract from and reduce pain, particularly when that task is cognitively demanding [see Refs. 35 and 36]. This reduction in pain (*task-induced hypoalgesia*) suggests that task demands are a central factor in distraction. However, experimental evidence showing how motivation and task demands work together to reduce pain is limited.

Resource-based models of attention^14,28^ suggest that pain perception and task performance compete for access to a shared pool of attentional or cognitive resources [eg, Ref. 17, see also Ref. 26]. The capacity of this pool is limited, which necessitates that the resources be strategically allocated between processes. When a task's demands reach the pool's capacity, engaging in that task would leave few resources available for perceiving pain.^17,20,31^ However, a demanding task may not fully occupy these resources if the motivation to engage in the task is low. Indeed, people generally appear less motivated to engage in effortful cognitive tasks^43^ and will sometimes prefer to experience pain.^42^ This suggests that in previous studies on distraction, participants' motivation to engage in the task may have been suboptimal to produce the greatest pain-reducing effect.

We therefore tested the combined effects of motivation and task demands on pain and task performance by offering monetary rewards during a competing task. Previous studies have shown that offering monetary incentives leads to greater investment of cognitive resources into the task at hand^5,22,27^; thus, we predicted greater reductions in pain when the task was both rewarded and demanding. By contrast, we predicted that an easier task would be less affected by rewards because performance would be at a ceiling and thus would not benefit from additional investment of resources.^39^

Previous studies using rewards to examine task-induced hypoalgesia [eg,Ref. 40] held task demands constant across reward conditions and therefore are unable to specify if rewards interact with task demands to influence pain. Rewards may increase resources invested in the task, thereby amplifying the effects of task demands or alternatively may simply provide an additive hedonic effect that is unaffected by task demands.^3,11^ Here, we proposed that rewards would strengthen the effects of task-induced hypoalgesia, but only when task demands were high enough to allow performance to benefit from investing more cognitive resources into the task. By contrast, rewards would have limited effects on pain when task demands were minimal because investing more cognitive resources in the easy task would have limited benefits on performance.

To test this proposal, we used a within-subjects factorial design wherein we offered monetary rewards to perform a highly demanding 2-back task or a minimally demanding arrow discrimination task^35^ while receiving painful and nonpainful heat stimuli. We hypothesized that rewards would amplify the pain-reducing effects of the demanding task but have limited effects on the easier task. In line with this, we also aimed to test the effects of rewards on task performance. We predicted that 2-back performance would be higher on reward trials, but that rewards would have no effects on the easier arrow discrimination task because performance was expected to be near perfect. We also used multilevel mediation to further examine the relationship between task difficulty, performance, and pain,^7,35^ allowing us to see where and how rewards exerted their influence on pain and performance. We predicted that task performance would mediate the hypoalgesic effects of rewards^35^ such that rewards would increase performance, which in turn would reduce pain ratings.

## 2. Methods

### 2.1. Participants

Sixty pain-free adults (49 women and 11 men, aged 18–35 years, M = 21.5, SD = 3.0) were recruited through the McGill University community to take part in the study. Participants were ineligible for the study if they exhibited any of the following: previous or current diagnosis of chronic pain syndrome or neuropathy; psychiatric or neurological disorder; history of alcohol or other substance use; or use of analgesics, anticonvulsants, narcotics, antidepressants, or anxiolytics more than twice weekly. Data from 3 participants were excluded from analysis because of their failure to follow task instructions (performed the control task incorrectly); analyses were conducted on the remaining 57 participants (48 women and 9 men, aged 18–35 years, M = 21.4, SD = 3.1). Participants were compensated for their time with psychology course credits, if applicable, and at the rate of $15 CAD (Canadian dollars) per hour, in addition to any bonus winnings that they could earn (see “Monetary Incentives Calibration” section below). The McGill University Research Ethics Board approved the study, and each participant gave informed written consent before participating.

### 2.2. Thermal sensitivity calibration

We used a 9-cm^2^ thermal contact probe (TSA-II Neurosensory Analyzer; Medoc Ltd. Advanced Medical Systems, Israel) to apply heat stimuli throughout the experiment. To control for differences in thermal sensitivity, the temperatures were calibrated to each individual using the method of constant stimuli. To do this, we applied 7 different temperatures (40, 44, 45, 46, 47, 48, and 49°C), once each to 4 different sites on the volar surface of the participant's left forearm for a total of 28 stimulations. The temperature started at a 32°C baseline before increasing to the target temperature and returning to baseline (2.5-second rise time, 8-second plateau, and 2.5-second fall time). After each stimulation, participants indicated the temperature as either “not painful” or “painful” by pressing the left and right mouse buttons, respectively, and by providing ratings using a visual analogue scale. If participants indicated the temperature as nonpainful, they rated the warmth of the stimulus from 0 (*no warmth at all*) to 100 (*very hot, without pain*). If participants indicated the temperature as painful, they first rated its intensity from 0 (*not intense at all*) to 100 (*extremely intense*) and then rated its unpleasantness from 0 (*not unpleasant at all*) to 100 (*extremely unpleasant*). These ratings were then input to a regression model that adjusted the ratings based on central and peripheral adaptation processes [for details, see Ref. 13]. This model produced a temperature–response curve that allowed us to calculate a low, nonpainful temperature (corresponding to 80/100 on a warmth scale) and a high, painful temperature (60/100 on a pain scale) to use for each participant in the study. Across all participants, the average low temperature was 45.3°C (SD = 1.9) and the average high temperature was 48.4°C (SD = 0.6).

### 2.3. Two-back task calibration

**Figure 1.**
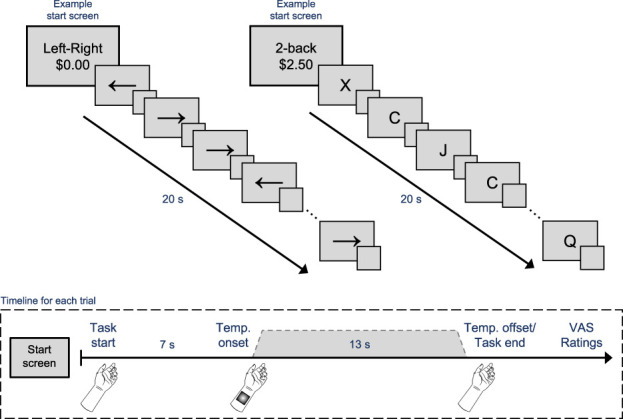
Examples of the motivated distraction task. Participants first saw which task to perform—the 2-back or the left–right arrow task—and the possible monetary reward for that trial. Half of the trials presented the calibrated reward amount, and the other half presented $0.00. Participants began the cognitive task, and after 7 seconds, either the low or the high temperature was applied to the participant's forearm (see timeline box). After the task ended and the temperature returned to baseline, participants rated their perception of the temperature using visual analogue scales (VASs).

To distract from the painful temperatures, we used the *N*-back working memory task. The *N*-back task presents a series of letters, and for each letter, participants are asked to respond whether the current letter is the “same” or “different” from the letter that was presented *N* letters before. We used the 2-back version for this study, wherein participants responded to letters that were presented 2 letters before the current letter (Fig. 1). To account for differences in cognitive ability, we calibrated the speed of the task to each person by adjusting the interstimulus interval (ISI)—the delay between presented letters (min: 19 ms, max: 2,583 ms).^7,35,42^ In each trial, a fixation cross was first presented for 250 ms, after which a letter character was presented for 500 ms. After the letter, a blank screen remained for the duration of the ISI. Participants could respond at any point after the letter was presented and before the next fixation cross appeared. This process repeated for 20 seconds, with the total number of letters presented based on the ISI.

This calibration procedure used a staircase method, wherein participants performed 15 trials of the 2-back task, with the ISI updating after each trial to obtain a level of performance between 0.75 and 0.85. To measure task performance, we used the nonparametric sensitivity measure “*A*”—an alternative to the common sensitivity measure *d′* used in signal detection theory—because it allows hit and false alarm rates to be at or near 0 or 1.^45^ Obtaining a score of 1 indicates perfect performance, a score of 0.5 indicates performance at chance level, and a score of 0 indicates all responses are incorrect. The average of the calibrated ISI across participants was 881.0 ms (SD = 395.6, minimum = 303, maximum = 2,583).

### 2.4. Monetary incentives calibration

Drawing from Fields' motivation–decision model,^10^ which argues that anything more important than pain will inhibit pain, we aimed to make the cognitive task more “important” than pain by ascribing it a monetary value that was higher than pain's value.^30,41^ As individuals differ markedly in how they value monetary rewards in the presence of pain,^8,30,41^ participants also completed a method of constant stimuli procedure to adjust the amount of money offered during the main experimental task. Here we offered participants 26 choices to receive a painful temperature (60/100 on a pain scale) for a variable amount of money ($0.00 to $3.00 CAD). Participants were told that if they accepted an offer, they would receive the painful temperature and could win the given amount at the end of the experiment. If they rejected an offer, they would avoid the painful temperature but lose the chance to win that money at the end of the experiment. Participants were not made aware of the exact probabilities of winning rewards but were simply told that they had a chance to win the given reward on a random draw of trials at the end of the experiment. In reality, their choices determined the amount of money displayed during the following motivated distraction task (see below).

After all choices were made, their decisions were input into a logistic regression model that calculated the amount of money that each participant would accept 95% of the time—ie, the amount of money necessary for the person to say “yes” to the pain-for-money offer 95% of the time. The average of this final calculated amount across participants was $2.02 (SD = $1.04), with the minimum and maximum allowed offers being $0.50 and $3.00 CAD, respectively.

### 2.5. Motivated distraction task

For the main experimental task, participants performed a computerized task while receiving thermal stimulations [see Refs 7 and 35]. At the start of each trial, the to-be-performed task was presented alongside a monetary figure—either $0.00 or the amount determined from the monetary incentive calibration (Fig. 1). Participants were instructed that for good performance, they would be given the chance to win this amount of money at the end of the experiment. This was done to motivate people to fully engage in and perform well on the task,^5,40^ as well as avoid any potential negative affect resulting from failing to meet the required level of performance. At the end of the experiment, participants were awarded 10% of the maximum amount they could win (calibrated reward amount × 32 trials × 10%) regardless of their actual performance. Participants performed either a highly demanding 2-back task or a minimally demanding left–right (LR) arrow task. The LR arrow task was used as the control task because its working memory demands are minimal but controls for the motor demands of the task as well as focusing attention externally. In this, participants were asked to respond to the direction of left and right arrows using the left and right mouse buttons, respectively, but were not required to remember previously shown arrows. Instead, they simply responded to the most recent arrow. The same ISI was used for both the 2-back and the LR task.

Finally, one of 2 temperatures was applied to the participants' left forearm: a low, nonpainful, warm temperature (80/100 on a warmth scale) or a high, painful temperature (60/100 on a pain scale). The temperature was applied 7 seconds after the start of the trial and returned to baseline when the task ended (2.5-second rise time, 8-second plateau, and 2.5-second fall time). The participants would then rate their perception of the thermal stimulus. Specifically, they indicated the temperature as either “not painful” or “painful” and provided ratings using a visual analogue scale. If they indicated the temperature as nonpainful, they rated the stimulus from 0 (*no warmth at all*) to 100 (*very hot, without pain*), and if they indicated the temperature as painful, they first rated its intensity from 0 (*not intense at all*) to 100 (*extremely intense*) and then its unpleasantness from 0 (*not unpleasant at all*) to 100 (*extremely unpleasant*). Each trial lasted 20 seconds and was presented in a pseudo-random fashion. Participants completed 64 trials in total and were unaware of the specific temperature being applied.

### 2.6. Procedure

The study consisted of a single 3-hour session. Participants were first welcomed and informed of the procedures of the study. After providing written consent, participants did the following procedures: thermal sensitivity calibration, 2-back task calibration, completed questionnaires measuring individual differences, monetary incentives calibration, and the motivated distraction task. The calibration procedures allowed us to examine the impact of individual traits beyond differences in pain sensitivity, monetary sensitivity, or cognitive ability. After all tasks were complete, each person was debriefed, awarded their winnings, and compensated for their time.

### 2.7. Questionnaire-based measures

To explore potential individual differences in motivated distraction, participants completed questionnaires measuring pain catastrophizing, trait mindfulness, trait anxiety, and state anxiety. Specifically, participants completed the Pain Catastrophizing Scale,^33^ Five-Facet Mindfulness Questionnaire,^1^ and the State–Trait Anxiety Inventory.^32^ Our preliminary analyses showed no significant influence of these measures on our effects of interest (see Supplementary Material for analyses of these measures, available at http://links.lww.com/PR9/A174).

### 2.8. Data analyses

We performed statistical analyses with R version 4.0.5. We used the “afex” package^29^ to conduct the analysis of variances (ANOVAs) comparing thermal ratings and task performance between the different conditions. Planned contrasts of the effects were conducted using the “emmeans” package^19^; the Holm–Bonferroni method was used to adjust for multiple comparisons. To test our main hypothesis of an interaction between motivation and task demands, we conducted a 2 × 2 repeated-measures ANOVA comparing pain ratings between the 2 tasks (LR vs 2-back) and reward conditions (reward vs no reward). As our hypotheses related to the effects of distraction on pain perception specifically, we only analyzed those trials in which the high, painful temperature was presented.^35^ If the participant indicated the temperature as nonpainful, their rating was converted to a 0 on the pain rating scale. Trials where temperature ratings were provided faster than 150 ms were removed from analysis (1.1% of trials). As pain perception and task performance seem to be inversely related,^7^ we also conducted a 2 × 2 repeated-measures ANOVA to compare 2-back performance between the heat level (low vs high) and reward conditions (reward vs no reward). As performance was expectedly at ceiling for the LR task, we analyzed only trials in which the 2-back was performed to examine any changes in performance based on the interaction between heat level and rewards.^35^

We also used a multilevel mediation analysis to further assess the role of rewards and task demands in task-induced hypoalgesia. This allowed us to capture trial-by-trial fluctuations between task performance and pain perception within the same reward or task demand conditions while also accounting for participant-level differences between them. Specifically, we examined whether task performance mediated the relationship between task demands and pain perception, and how rewards moderated these effects. We controlled for time-based effects by including trial number and log-transformed trial number as covariates in the model. We used the “nlme” package^23^ to perform the analysis using the approach advocated by Bauer et al.^2^ Random effects were estimated for the *a*, *b*, and *c′* paths using restricted maximum likelihood. (In what follows, we use *ab* as shorthand for the indirect effect. The Bauer et al. model includes the covariance between the random effects of the *a* and *b* paths in their computation of the indirect effect, and thus is more than the simple product of these coefficients.) Pain ratings were rescaled from a 100-point scale to a 0 to 1 scale to reduce converge errors in fitting the model. Custom code was used to obtain the bootstrapped distributions (10,000 replications, resampling at the participant level)^16^ of the indirect effects and percentile confidence intervals reported below and is available at https://github.com/falkcarl/multilevelmediation.

## 3. Results

### 3.1. Changes in pain ratings

**Figure 2.**
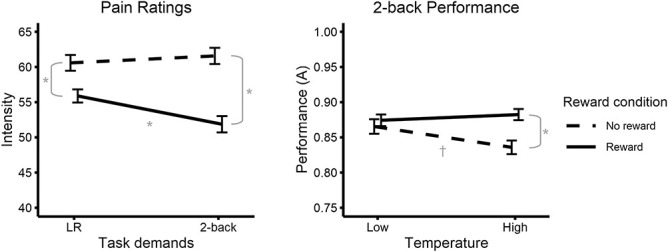
Pain and performance interactions with rewards. (A) Effect of task demands and rewards on thermal ratings for the high temperature. (B) Effect of temperature and rewards on 2-back performance. Error bars are within-subject SE using the Cousineau–O’Brien^9^ method to remove between-subjects differences. LR: Left–right arrow discrimination task; †*P* = 0.06, *P < 0.05.

As predicted, we observed a significant interaction between task demands and monetary rewards (Fig. 2A), such that pain intensity ratings were lower when performing the 2-back task relative to the LR task, but only when rewards were offered (*F*(1, 56) = 8.59, *P* = 0.005, ${\text{η}}_{p}^{2}$ = 0.13). There was a significant main effect of rewards on pain ratings (*F*(1, 56) = 41.76, *P* < 0.001, ${\text{η}}_{p}^{2}$ = 0.43), but there was no significant main effect of task demands (*F*(1, 56) = 1.40, *P* = 0.24, ${\text{η}}_{p}^{2}$ = 0.02). Planned contrast analyses showed that, when rewards were offered, pain was significantly lower during the 2-back relative to the LR task, *t*(56) = 2.78, *P* = 0.015). When no rewards were offered, there was no significant difference in pain between the 2-back and LR task (*t*(56) = 0.61, *P* = 0.55). During the 2-back, pain was significantly lower when rewards were offered relative to no rewards (*t*(56) = 6.29, *P* < 0.001). Finally, pain was significantly lower during the LR task when rewards were presented relative to when no rewards were presented (*t*(56) = 3.76, *P* = 0.001).

Ratings of pain unpleasantness showed similar results to that of pain intensity. There was a significant interaction between task demands and rewards showing that unpleasantness ratings were lower during the 2-back relative to the LR task, but only when rewards were offered (*F*(1, 56) = 5.87, *P* = 0.019, ${\text{η}}_{p}^{2}$ = 0.09). However, planned contrast analyses showed no significant differences between the LR and 2-back conditions when rewards were presented (*t*(56) = 1.42, *P* = 0.32) nor when rewards were absent (*t*(56) = 1.23, *P* = 0.32). Pain unpleasantness was significantly lower when rewards were presented during the 2-back (*t*(56) = 6.44, *P* < 0.001) and the LR task (*t*(56) = 4.53, *P* < 0.001) relative to when no rewards were presented, respectively.

### 3.2. Changes in task performance

We also examined whether pain disrupted participants' performance of the 2-back task, and whether rewards reduced this disruption. Mirroring the effects on pain ratings, we observed that 2-back performance was lowest during high heat (painful) trials, but that rewards mitigated this effect. That is, a significant interaction between heat level and rewards (*F*(1, 56) = 6.33, *P* = 0.015, ${\text{η}}_{p}^{2}$ = 0.10; Figure 2b) showed that performance declined during high heat trials when rewards were absent (*t*(56) = 2.37, *P* = 0.064), but that performance was maintained when rewards were offered (*t*(56) = 1.11, *P* = 0.54). We found no main effect of heat level (*F*(1, 56) = 2.38, *P* = 0.13, ${\text{η}}_{p}^{2}$ = 0.04), but did find a significant main effect of rewards (*F*(1, 56) = 5.36, *P* = 0.024, ${\text{η}}_{p}^{2}$ = 0.09) on performance. On low temperature trials, there was no significant difference in performance between the 2 reward conditions (*t*(56) = 0.61, *P* = 0.55), whereas performance was significantly greater in high temperature trials when rewards were presented (*t*(56) = 3.41, *P* = 0.005.

### 3.3. Mediating effects of task performance on pain

**Figure 3.**
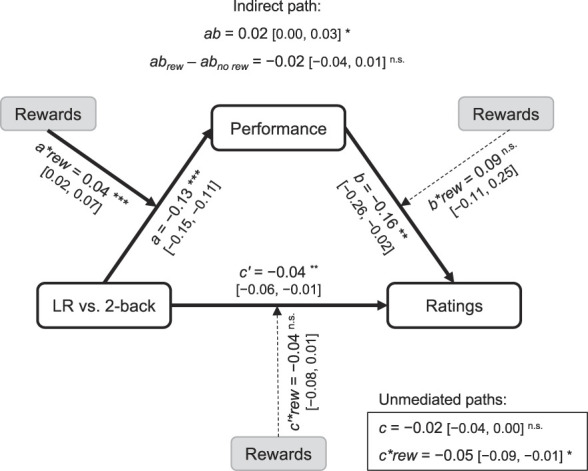
Path diagram for mediation of task demands on thermal ratings. Performance dropped when performing the demanding 2-back task (*a* path), but rewards helped maintain performance despite the higher difficulty (a*rew path). Higher performance was associated with lower pain ratings (*b* path), reflecting an inverse relationship between them. There was an indirect effect of task demands on pain through changes in performance (*ab* path). That is, when performance was higher, pain ratings were lower, but maintaining good performance during the 2-back is more challenging. Thus, there was a small anti-hypoalgesic effect of the higher difficulty of the 2-back, which lessened its pain-reducing effect. Rewards did not moderate any other paths in the model, suggesting that rewards operate by influencing the motivation to engage in the task and perform well. *P < 0.05, **P < 0.01, ***P < 0.001.

Our central hypothesis argues that rewards strengthen the effect of distraction by motivating people to engage and perform well in the demanding task, thereby allocating more attentional resources away from pain. Extending this, we also predicted that (1) pain would be lower in trials where performance was high and (2) performance would increase with rewards, thereby indirectly reducing pain. To test this, we conducted a multilevel mediation analysis^7,35^ to examine the trial-by-trial relationship between task demands, performance, rewards, and pain. Specifically, we sought to examine whether task performance mediated the relationship between task demands and pain, and further, if rewards moderated these relationships (Fig. 3). First examining the total effect of task demands on pain ratings, not accounting for performance, we found a nonsignificant effect of task demands (*c* path, total effect, *c* = −0.02, 95% confidence limit [CI] [−0.04 to 0.00], *t*(1756) = 1.80, *P* = 0.073) that was significantly moderated by rewards (c*rew = −0.05, 95% CI [−0.09 to −0.01], *t*(1756) = 2.43, *P* = 0.015), reflecting the interaction between task demands and rewards shown in the ANOVAs above.

There was a significant effect of task demands on performance (*a* path), wherein performance was lower for the 2-back relative to the LR task (*a* = −0.13, 95% CI [−0.15 to −0.11], *t*(3566) = 10.92, *P* < 0.001). There was also a significant effect of performance on pain ratings (*b* path), wherein higher levels of performance corresponded with lower pain (*b* = −0.16, 95% CI [−0.26 to −0.02], *t*(3566) = 3.23, *P* = 0.001). In line with this, there was a significant indirect effect (*ab* path) of task demands on pain ratings through changes in performance (*ab* = 0.02, 95% CI [0.00–0.03]). There was also a significant direct effect of task demands on pain, after controlling for performance (*c′* path), showing that the 2-back task reduces pain regardless of performance (*c′* = −0.04, 95% CI [−0.06 to −0.01], *t*(3566) = 3.06, *P* = 0.002). The combined direct and indirect effects indicated that, when performance on the 2-back was low, the pain-reducing effects of distraction were weaker. Conversely, when performance in the 2-back task was high, this led to stronger hypoalgesia. Finally, there was a significant effect of log-transformed trial number, which was independent of task design, which indicated the presence of rapid pain sensitization over the first trials of the task (*log*(trial number) = 0.03, 95% CI [0.00–0.06], *t*(3566) = 2.59, *P* = 0.010). Examining how rewards moderated these effects, we found that rewards (*rew*) significantly moderated the relationship between task demands and performance, wherein 2-back performance during pain was maintained when rewards were presented (a*rew = 0.04, 95% CI [0.02–0.07], *t*(3566) = 4.16, *P* < 0.001). Rewards did not significantly moderate any of the other paths (Table 1).

**Table 1:** Estimates from mediation model on ratings of high temperature trials.

Predictor	Estimate	95% confidence limits		DF	*t*	*P*
		Lower	Upper			
Outcome: performance						
(Intercept)	0.96	0.93	0.99	3566	80.22	<0.001***
Task demands	−0.13	−0.15	−0.11	3566	10.92	<0.001***
Reward	0.00	−0.00	0.01	3566	0.32	0.75
Task demands × reward	0.04	0.02	0.07	3566	4.16	<0.001***
Trial number	0.00	−0.00	0.00	3566	0.14	0.89
*log*(trial number)	0.01	−0.01	0.02	3566	1.04	0.30
Outcome: ratings						
(Intercept)	0.64	0.48	0.76	3566	10.73	<0.001***
Performance	−0.16	−0.26	−0.02	3566	3.23	0.001**
Reward	−0.13	−0.29	0.07	3566	1.46	0.15
Performance × reward	0.09	−0.11	0.25	3566	1.00	0.32
Task demands	−0.04	−0.06	−0.01	3566	3.06	0.002*
Task demands × reward	−0.04	−0.08	0.01	3566	1.46	0.14
Trial number	−0.00	−0.00	0.00	3566	0.13	0.90
*log*(trial number)	0.03	0.00	0.06	3566	2.59	0.010**
Indirect effect	0.02	0.00	0.03*			
Difference of indirect effects	−0.02	−0.04	0.01			

*P < .05, **P < .01, ***P < .001.

## 4. Discussion

Although pain naturally grabs attention, engaging in a competing task can distract from and inhibit the experience of pain. One important determinant of the task's ability to reduce pain seems to be its demands: more cognitively demanding tasks tend to produce greater hypoalgesia compared with minimally demanding tasks [for reviews, see Refs. 35 and 36]. Considering that pain always occurs in the context of other ongoing activities, it could be said that consciously perceived pain is a function of the relative importance of pain with respect to other goals. That is, when the motivation to attend to pain is less than that of a competing alternative, pain will be inhibited to allow more important actions to continue.^10^ The current study provides evidence that engaging in a cognitive task can reduce pain, but that this hypoalgesic effect is influenced by both the demands of the task and the person's motivation to engage in the task.

These findings support limited resource-based models of distraction,^17,20^ whereby engaging in a competing task reduces the availability of resources to attend to pain. These cognitive resources are not necessarily depleted or consumed by the task but rather allocated towards one process over another. Here, the degree of hypoalgesia is proportional to the level of resources invested in the task. If the task is minimally demanding, motivation will have limited effects on reducing pain because performance is already at maximum and thus investing more resources in the task would be wasteful. These resources therefore are left available for capture by pain, even though motivation to complete the task may be high. Similarly, high task demands will have little impact on pain if motivation is low; a demanding cognitive task can reduce feelings of pain, but only if the person is willing to invest cognitive resources in the task. Here, we observed a supra-additive interaction between task demands and motivation such that the combination of the 2 is necessary to obtain optimal pain reduction.

Interestingly, these findings also align well with predictions derived from Brehm's motivational intensity theory.^6,24^ This theory argues that the amount of effort (ie, cognitive resources) invested in a task is determined by the task's difficulty and importance. Although task performance is an indirect measure of effort mobilization, we found that pain was lowest when performance was high (*b* path, mediation model), suggesting that pain perception reliably tracked effort mobilization through engagement and performance of the 2-back task. Similar effects were also found when examining how pain interferes with task performance: rewards increased the motivation to invest effort into the task, which prevented pain from disrupting performance of the demanding task.

Finally, an analysis of trial-by-trial fluctuations between pain and task performance showed that rewards increased the hypoalgesic effects of a demanding cognitive task through changes in task performance. Specifically, a multilevel mediation analysis revealed that increasing task demands had a direct hypoalgesic effect on pain ratings (*c′* path) and that higher task performance led to less pain (*b* path). However, this pain-reducing effect of high task demands seemed to be partly suppressed by the increased difficulty of the task. That is, maintaining good performance is harder during the 2-back task, which leads to an anti-hypoalgesic effect related to task performance (positive *ab* mediation term). High task demands cause a decrease in performance (negative *a* path), which in turn produce an indirect increase in pain (negative *b* path). Monetary rewards reduced the impact of this indirect anti-hypoalgesic effect by preventing performance from declining despite the task demands (a*rew path). In other words, the results of our multilevel mediation model suggest that rewards motivated participants to maintain a high level of performance in the distracting task despite the increased difficulty, and that this amplified the pain-reducing effects of the task.

The present findings also expand on previous reports of rewards and pain^4,40^ and describe an important mechanism in how rewards can reduce pain: increasing the amount of cognitive resources allocated towards a competing task. However, there was a significant, albeit smaller, effect of rewards on pain during the minimally demanding arrow discrimination task. This effect of rewards during the easier task, however, can still be interpreted as reflecting increased engagement in the task, although ceiling effects may have prevented this effect from being manifested as increased task performance. Alternatively, the presence of a significant hypoalgesic effect of rewards in the control condition may also be reflective of an additional hedonic effect of rewards, one that is independent from the reallocation of attentional resources towards a competing task.^4,18^ However, our key finding of an interaction between rewards and task demands rules out a purely hedonic effect of rewards on pain. Future studies should aim at disentangling the effects of rewards from task demands, for example, by specifying whether the presence of a cognitive task is always necessary for observing pain-reducing effects of rewards delivered at the same time as pain.

In contrast to previous studies that used similar distraction paradigms without rewards,^7,25,35^ we did not observe a main effect of task demands on pain ratings during the no-reward trials. Here, we believe that participants strategically reserved their resources during no-reward trials so as to maximize their chances of success during the reward trials. Previous research has shown that people perform worse when they anticipate a demanding task in the near future compared with not anticipating a task,^21^ suggesting that people will conserve their resources to avoid overexerting themselves. This strategic allocation would therefore lead participants to hold back during nonrewarded trials, making the distracting task less effective at reducing pain.

One limitation of our study is that we only tested 2 levels of difficulty for the cognitive task. Based on our findings, we would expect that increasing the difficulty of the task would lead to even greater reductions in pain when rewards are present. However, increasing the difficulty is likely effective only up to a certain point, after which no more resources would be available to invest in the task, or perhaps people would perceive the task as too difficult and would disengage. Future studies should aim to test how increasing task difficulty may lead to greater hypoalgesia and whether there is a “breaking point” after which increasing difficulties become less effective at reducing pain.

More broadly, the present findings are compatible with 2 prominent models of pain–cognition interactions. First, our results align well with Fields'^10^ motivation–decision model, which posits that any activity more important than pain will block pain through brainstem circuits affecting the transmission of ascending nociceptive signals at the spinal level. However, Fields'^10^ model solely focuses on competing motivations and does not account for the demands of the competing actions. In contrast, neurocognitive model by Legrain et al.^17^ argues that pain is prevented from entering awareness based on 2 factors: goal-relevant information held in working memory and the amount of attention deployed towards the task. However, the original formulation of the model neglected motivational factors. Although later models^36,38^ incorporated motivational^37^ and emotional^25,34^ factors, experimental evidence demonstrating how motivation interacted with a competing task was missing.

The present findings therefore bridge the motivation–decision and neurocognitive models of pain–cognition interactions by showing that both high task demands and motivation are necessary to inhibit pain when completing a competing cognitive task. These findings also extend previous literature by identifying factors (task demands, task engagement, resource allocation, etc.) that can be manipulated to optimize pain inhibition. To maximize experimental control over these factors, we chose to use extrinsic monetary rewards to increase the motivation to perform an intrinsically unpleasant cognitive task.^42^ However, we can presume that motivation to engage in the tasks in the present study may have still been suboptimal and that more intrinsically rewarding tasks such as video games may have produced even greater reductions in pain.^12,44^ Altogether, the present findings demonstrate that subjectively perceived pain depends on the relative investment of attentional resources between pain processing and other valued goals, and that these effects are dynamic and potentially substantial. Therefore, learning to harness and optimize the pain-reducing effects of engaging and rewarding activities could have important implications for pain management.

## Disclosures

The authors have no conflicts of interest to declare.

## Appendix A. Supplemental digital content

Supplemental digital content associated with this article can be found online at http://links.lww.com/PR9/A174.

## Supplementary Material

SUPPLEMENTARY MATERIAL

## References

[1] Baer RA Smith GT Hopkins J Krietemeyer J Toney L. Using self-report assessment methods to explore facets of mindfulness. Assessment 2006;13:27–45. 16443717 10.1177/1073191105283504

[2] Bauer DJ Preacher KJ Gil KM. Conceptualizing and testing random indirect effects and moderated mediation in multilevel models: new procedures and recommendations. Psychol Methods 2006;11:142–63. 16784335 10.1037/1082-989X.11.2.142

[3] Becker S Gandhi W Elfassy NM Schweinhardt P. The role of dopamine in the perceptual modulation of nociceptive stimuli by monetary wins or losses. Eur J Neurosci 2013;38:3080–8. 23841460 10.1111/ejn.12303

[4] Becker S Gandhi W Pomares F Wager TD Schweinhardt P. Orbitofrontal cortex mediates pain inhibition by monetary reward. Soc Cogn Affect Neurosci 2017;12:651–61. 28119505 10.1093/scan/nsw173 PMC5390724

[5] Botvinick M Braver T. Motivation and cognitive control: from behavior to neural mechanism. Annu Rev Psychol 2015;66:83–113. 25251491 10.1146/annurev-psych-010814-015044

[6] Brehm JW Self EA. The intensity of motivation. Annu Rev Psychol 1989;40:109–31. 2648973 10.1146/annurev.ps.40.020189.000545

[7] Buhle J Wager TD. Performance-dependent inhibition of pain by an executive working memory task. PAIN 2010;149:19–26. 20129735 10.1016/j.pain.2009.10.027 PMC4229048

[8] Coll MP Slimani H Woo CW Wager TD, Rainville P Vachon-Presseau É, Roy M. The neural signature of the decision value of future pain. Proc Natl Acad Sci 2022;119:e2119931119. 35658082 10.1073/pnas.2119931119 PMC9191656

[9] Cousineau D O'Brien F. Error bars in within-subject designs: a comment on Baguley (2012). Behav Res Methods 2014;46:1149–51. 24477859 10.3758/s13428-013-0441-z

[10] Fields HL. A motivation-decision model of pain: the role of opioids. Proceedings of the 11th World Congress on Pain, 2006:449–59.

[11] Fields HL. Understanding how opioids contribute to reward and analgesia. Reg Anesth Pain Med 2007;32:242–6. 17543821 10.1016/j.rapm.2007.01.001

[12] Jameson E Trevena J Swain N. Electronic gaming as pain distraction. Pain Res Manag 2011;16:27–32. 21369538 10.1155/2011/856014 PMC3052404

[13] Jepma M Jones M Wager TD. The dynamics of pain: evidence for simultaneous site-specific habituation and site-nonspecific sensitization in thermal pain. J Pain 2014;15:734–46. 24768695 10.1016/j.jpain.2014.02.010 PMC4083082

[14] Kahneman D. Attention and effort. Englewood Cliffs, NJ: Prentice-Hall, 1973. Available at: https://www.jstor.org/stable/1421603?origin=crossref.

[15] Klein C. What the body commands: the imperative theory of pain. Cambridge: The MIT Press, 2015.

[16] van der Leeden R Meijer E Busing FMTA. Resampling multilevel models. In: de Leeuw J Meijer E, eds. Handbook of Multilevel Analysis. New York: Springer, 2008; 401–33.

[17] Legrain V Van Damme S Eccleston C Davis KD Seminowicz DA Crombez G. A neurocognitive model of attention to pain: behavioral and neuroimaging evidence. PAIN 2009;144:230–2. 19376654 10.1016/j.pain.2009.03.020

[18] Leknes S Tracey I. A common neurobiology for pain and pleasure. Nat Rev Neurosci 2008;9:314–20. 18354400 10.1038/nrn2333

[19] Lenth RV. emmeans: Estimated marginal means, aka least-squares means, 2021. Available at: https://CRAN.R-project.org/package=emmeans. Accessed November 10, 2021.

[20] McCaul KD Malott JM. Distraction and coping with pain. Psychol Bull 1984;95:516–33. 6399756

[21] Muraven M Shmueli D Burkley E. Conserving self-control strength. J Pers Soc Psychol 2006;91:524–37. 16938035 10.1037/0022-3514.91.3.524

[22] Otto AR Vassena E. It's all relative: reward-induced cognitive control modulation depends on context. J Exp Psychol Gen 2021;150:306–13. 32790463 10.1037/xge0000842

[23] Pinheiro J Bates D DebRoy S Sarkar D; R Core Team. nlme: Linear and nonlinear mixed effects models, 2021. Available at: https://CRAN.R-project.org/package=nlme. Accessed November 10, 2021.

[24] Richter M Gendolla GHE Wright RA. Three decades of research on motivational intensity theory. Adv Motiv Sci 2016;3:149–86. doi:

[25] Rischer KM González-Roldán AM Montoya P Gigl S Anton F Meulen M. Distraction from pain: the role of selective attention and pain catastrophizing. Eur J Pain 2020;24:1880–91. 32677265 10.1002/ejp.1634 PMC7689692

[26] Shenhav A Musslick S Lieder F Kool W Griffiths TL Cohen JD Botvinick MM. Toward a rational and mechanistic account of mental effort. Annu Rev Neurosci 2017;40:99–124. 28375769 10.1146/annurev-neuro-072116-031526

[27] Shenhav A Prater Fahey M Grahek I. Decomposing the motivation to exert mental effort. Curr Dir Psychol Sci 2021;30:307–14. 34675454 10.1177/09637214211009510 PMC8528169

[28] Shiffrin RM Schneider W. Controlled and automatic human information processing: II. Perceptual learning, automatic attending and a general theory. Psychol Rev 1977;84:127–90.

[29] Singmann H Bolker B Westfall J Aust F Ben-Shachar MS. afex: Analysis of factorial experiments, 2021. Available at: https://CRAN.R-project.org/package=afex. Accessed November 10, 2021.

[30] Slimani H Rainville P Roy M. The aversive value of pain in human decision-making. Eur J Pain 2022;26:668–79. 34845800 10.1002/ejp.1895

[31] Sörqvist P Marsh JE. How concentration shields against distraction. Curr Dir Psychol Sci 2015;24:267–72. 26300594 10.1177/0963721415577356 PMC4536538

[32] Spielberger CD. State-trait anxiety inventory. In: Weiner IB Craighead WE, eds. The Corsini Encyclopedia of Psychology. Hoboken: John Wiley & Sons, Inc, 2010; corpsy0943.

[33] Sullivan MJL Bishop SR Pivik J. The pain catastrophizing scale: development and validation. Psychol Assess 1995;7:524–32.

[34] Sussman TJ Szekely A Hajcak G Mohanty A. It's all in the anticipation: how perception of threat is enhanced in anxiety. Emotion 2016;16:320–7. 26479770 10.1037/emo0000098

[35] Tabry V Vogel TA Lussier M Brouillard P Buhle J Rainville P Bherer L Roy M. Inter-individual predictors of pain inhibition during performance of a competing cognitive task. Sci Rep 2020;10:1–14. 33311585 10.1038/s41598-020-78653-z PMC7732830

[36] Torta DM Legrain V Mouraux A Valentini E. Attention to pain! A neurocognitive perspective on attentional modulation of pain in neuroimaging studies. Cortex 2017;89:120–34. 28284849 10.1016/j.cortex.2017.01.010 PMC7617013

[37] Van Damme S Legrain V Vogt J Crombez G. Keeping pain in mind: a motivational account of attention to pain. Neurosci Biobehav Rev 2010;34:204–13. 19896002 10.1016/j.neubiorev.2009.01.005

[38] Van Ryckeghem DML Crombez G. Pain and attention: toward a motivational account. In: Motivational perspectives on chronic pain: theory, research, and practice. Oxford, United Kingdom: Oxford University Press, 2018; pp.211–46.

[39] Vassena E Deraeve J Alexander WH. Task-specific prioritization of reward and effort information: novel insights from behavior and computational modeling. Cogn Affect Behav Neurosci 2019;19:619–36. 30607834 10.3758/s13415-018-00685-w

[40] Verhoeven K Crombez G Eccleston C Van Ryckeghem DML Morley S Van Damme S. The role of motivation in distracting attention away from pain: an experimental study. PAIN 2010;149:229–34. 20188469 10.1016/j.pain.2010.01.019

[41] Vlaev I Seymour B Dolan RJ Chater N. The price of pain and the value of suffering. Psychol Sci 2009;20:309–17. 19254237 10.1111/j.1467-9280.2009.02304.x PMC2817525

[42] Vogel TA Savelson ZM Otto AR Roy M. Forced choices reveal a trade-off between cognitive effort and physical pain. eLife 2020;9:1–18. 10.7554/eLife.59410 PMC7714391 33200988

[43] Westbrook A Kester D Braver TS. What is the subjective cost of cognitive effort? Load, trait, and aging effects revealed by economic preference. PLoS One 2013;8:e68210. 23894295 10.1371/journal.pone.0068210 PMC3718823

[44] Wiederhold MD Wiederhold BK. Virtual reality and interactive simulation for pain distraction. Pain Med 2007;8:S182–8.

[45] Zhang J Mueller ST. A note on ROC analysis and non-parametric estimate of sensitivity. Psychometrika 2005;70:203–12.

