# Supplementary material for: Distraction from pain depends on task demands and motivation
Source: Pain Rep. 2022 Oct 26;7(6):e1041. doi: 10.1097/PR9.0000000000001041 (PMC9612955; doi:10.1097/PR9.0000000000001041)
Supplement: SUPPLEMENTARY MATERIAL [file painreports-7-e1041-s001.pdf]

Supplementary Material

Analyses of Individual Differences

Examining measures of individual differences we found no significant influence of pain catastrophizing, trait mindfulness, nor state- and trait-anxiety on pain ratings (see tables below). We examined each individual difference measure separately using a linear mixed-effects model to test whether the measure of interest moderated the relationship between task demands and rewards on pain ratings. For each measure, there was a significant interaction between task demands and rewards, as reported in the main text, even after controlling for individual differences. The lack of significant findings here likely reflects our calibration procedures used at the beginning of the experiment. These were designed to account for between-person differences in pain sensitivity and cognitive ability.

Supplementary Table 1

Moderating Effect of Pain Catastrophizing Scale

Effect	SS	MS	Num. DF	Den. DF	*F*	*p*
Task demands	1,100.2	1,100.2	1	1,755.01	2.06	.15
Reward	23,419.8	23,419.8	1	1,755.00	43.77	< .001 ***
PCS	1.6	1.6	1	54.99	0.00	.96
Task demands × Reward	2,948.7	2,948.7	1	1,755.00	5.51	.019 *
Task demands × PCS	516.3	516.3	1	1,755.01	0.96	.33
Reward × PCS	546.2	546.2	1	1,755.01	1.02	.31
Task demands × Reward × PCS	133.6	133.6	1	1,755.01	0.25	.62

Note. Type III Analysis of Variance table with Satterthwaite's method for degrees of freedom. PCS = Pain Catastrophizing Scale; SS = Sum of Squares; MS = Mean Square; * $p < .05$, *** $p < .001$.

Supplementary Table 2

Moderating Effect of Five-Facet Mindfulness Questionnaire

Effect	SS	MS	Num DF	Den DF	*F*	*p*
Task demands	943.0	943.0	1	1724.01	1.75	.19
Reward	24059.7	24059.7	1	1724.01	44.68	< .001 ***
FFMQ	6.6	6.6	1	53.97	0.01	.91
Task demands × Reward	2975.9	2975.9	1	1724.01	5.53	.019 *
Task demands × FFMQ	0.3	0.3	1	1723.99	0.00	.98
Reward × FFMQ	749.9	749.9	1	1723.99	1.39	.24
Task demands × Reward × FFMQ	1307.9	1307.9	1	1723.99	2.43	.12

Note. Type III Analysis of Variance table with Satterthwaite's method for degrees of freedom. FFMQ = Five-Facet Mindfulness Questionnaire; SS = Sum of Squares; MS = Mean Square; * $p < .05$, *** $p < .001$.

Supplementary Table 3

Moderating Effect of State Anxiety

Effect	SS	MS	Num DF	Den DF	*F*	*p*
Task demands	1,096.9	1,096.9	1	1,755	2.05	.15
Reward	23,405.5	23,405.5	1	1,755	43.73	< .001 ***
SAI	193.5	193.5	1	55	0.36	.55
Task demands × Reward	2,946.4	2,946.4	1	1,755	5.50	.019 *
Task demands × SAI	25.1	25.1	1	1,755	0.05	.83
Reward × SAI	695.6	695.6	1	1,755	1.30	.25
Task demands × Reward × SAI	97.9	97.9	1	1,755	0.18	.67

Note. Type III Analysis of Variance table with Satterthwaite's method for degrees of freedom. SAI = State–Trait Anxiety Inventory (State anxiety scale); SS = Sum of Squares; MS = Mean Square; * $p < .05$, *** $p < .001$.

Supplementary Table 4

Moderating Effect of Trait Anxiety

Effect	SS	MS	Num DF	Den DF	*F*	*p*
Task demands	1,093.1	1,093.1	1	1,755.01	2.04	.15
Reward	23,416.4	23,416.4	1	1,755.01	43.76	< .001 ***
TAI	44.9	44.9	1	55.01	0.08	.77
Task demands × Reward	2,944.6	2,944.6	1	1,755.01	5.50	.019 *
Task demands × TAI	61.7	61.7	1	1,755.03	0.12	.73
Reward × TAI	71.1	71.1	1	1,755.02	0.13	.72
Task demands × Reward × TAI	1,016.2	1,016.2	1	1,755.03	1.90	.17

Note. Type III Analysis of Variance table with Satterthwaite's method for degrees of freedom. TAI = State–Trait Anxiety Inventory (Trait anxiety scale); SS = Sum of Squares; MS = Mean Square; * $p < .05$, *** $p < .001$.
